# Dynamic changes of tumor gene expression during repeated pressurized intraperitoneal aerosol chemotherapy (PIPAC) in women with peritoneal cancer

**DOI:** 10.1186/s12885-016-2668-4

**Published:** 2016-08-19

**Authors:** Günther A. Rezniczek, Friederike Jüngst, Hendrik Jütte, Andrea Tannapfel, Ziad Hilal, Lukas A. Hefler, Marc-André Reymond, Clemens B. Tempfer

**Affiliations:** 1 Department of Obstetrics and Gynecology, Ruhr-Universität Bochum, Bochum, Germany; 2 Department of Pathology, Ruhr-Universität Bochum, Bochum, Germany; 3 Department of Obstetrics and Gynecology, Krankenhaus der Barmherzigen Schwestern, Linz, Austria; 4 Department of Surgery, Ruhr-Universität Bochum, Bochum, Germany; 5 Marien Hospital Herne, Düngelstr. 33, 44623 Herne, Germany; 6 Present Address: Department of General, Gastrointestinal and Transplantation Surgery, University of Tübingen, Tübingen, Germany

**Keywords:** Peritoneal cancer, Gene signature, Chemotherapy, PIPAC, Prognosis

## Abstract

**Background:**

Intraperitoneal chemotherapy is used to treat peritoneal cancer. The pattern of gene expression changes of peritoneal cancer during intraperitoneal chemotherapy has not been studied before. Pressurized intraperitoneal aerosol chemotherapy is a new form of intraperitoneal chemotherapy using repeated applications and allowing repeated tumor sampling during chemotherapy. Here, we present the analysis of gene expression changes during pressurized intraperitoneal aerosol chemotherapy with doxorubicin and cisplatin using a 22-gene panel.

**Methods:**

Total RNA was extracted from 152 PC samples obtained from 63 patients in up to six cycles of intraperitoneal chemotherapy. Quantitative real-time PCR was used to determine the gene expression levels. For select genes, immunohistochemistry was used to verify gene expression changes observed on the transcript level on the protein level. Observed (changes in) expression levels were correlated with clinical outcomes.

**Results:**

Gene expression profiles differed significantly between peritoneal cancer and non- peritoneal cancer samples and between ascites-producing and non ascites-producing peritoneal cancers. Changes of gene expression patterns during repeated intraperitoneal chemotherapy cycles were prognostic of overall survival, suggesting a molecular tumor response of peritoneal cancer. Specifically, downregulation of the whole gene panel during intraperitoneal chemotherapy was associated with better treatment response and survival.

**Conclusions:**

In summary, molecular changes of peritoneal cancer during pressurized intraperitoneal aerosol chemotherapy can be documented and may be used to refine individual treatment and prognostic estimations.

**Electronic supplementary material:**

The online version of this article (doi:10.1186/s12885-016-2668-4) contains supplementary material, which is available to authorized users.

## Background

Peritoneal carcinomatosis (PC) can occur in the form of primary peritoneal cancer or as a manifestation of a number of different malignancies such as ovarian, fallopian, colon, appendiceal, cholangiocellular, and gastric cancer [1, 2]. Irrespective of the source of origin, PC is difficult to treat and survival of affected patients is poor with a median duration of overall survival of 6–15 months [3–5]. Local and regional treatment strategies such as peritonectomy, peritonectomy combined with hyperthermic intraperitoneal chemoperfusion (HIPEC), and various modalities of intraperitoneal chemotherapy (IPC), including pressurized intraperitoneal aerosol chemotherapy (PIPAC), have been reported to achieve objective treatment responses in patients with PC of various origins [6–9]. In view of the poor prognosis of patients with PC, a better understanding of the molecular biology of this disease and the identification of new predictive and prognostic markers is an unmet medical need.

In patients with PC, distinct gene expression patterns of PC tumor cells have been associated with therapy response and prognosis. For example, Verhaak et al. used a 100 gene signature including RB1, NFKBIB, and RXRB for molecular subtyping of advanced ovarian cancer specimens with peritoneal metastases [10]. Others have used gene expression patterns to characterize the molecular pathway highlighting the transition of primary ovarian tumors to peritoneal metastases. For example, Brodsky et al. found that PC cells originating from ovarian cancer were more proliferative and less apoptotic than their respective primary tumors. In addition, peritoneal metastases had copy number aberrations that differed from those found in the primary tumor: a six gene expression signature including EFTUD1, CALB2, TIMP3, CYP1B1, IL7R, and RARRES2 distinguished primary from metastatic tumors and predicted overall survival [11]. In a similar study of 47 epithelial ovarian cancers, microarray analysis using an Affymetrix platform identified a 56 gene set with differential expression discriminating the primary tumor from peritoneal metastases [12]. Of note, 10/56 genes were involved in the p53 gene pathway. Matte et al. studied gene expression changes in human peritoneal mesothelial cells (HPMCs) exposed to malignant ascites from ovarian cancer with PC [13]. In this study, a total of 649 genes were differentially expressed in ascites-stimulated HPMCs with 484 genes up-regulated and 165 genes down-regulated. Thus, we felt it is reasonable to investigate whether tumor samples with contact to ascites would have different gene expression patterns compared to tumor samples without such exposure.

In summary, gene expression patterns in malignancies with PC such as ovarian cancer have prognostic and predictive value, discriminate between primary tumor and PC metastases, and react specifically to malignant ascites. A number of genes and gene pathways, e.g. p53, Akt, NF-kB, and VEGF seem to play a critical role in the development and sustained growth of PC. However, the pattern of gene expression changes of PC during chemotherapy has not been studied before. Whether or not gene expression changes of PC during chemotherapy have any prognostic or predictive value, is unknown. Here, we present the results of gene expression analyses of a panel of 22 genes in tissue samples obtained during repeated cycles of PIPAC in patients with PC originating from ovarian cancer. To our knowledge, this is the first study to investigate gene expression patterns during repeated applications of chemotherapy in patients with PC.

## Methods

### Patient samples

This is a retrospective analysis of samples and clinical data obtained during 152 IPC procedures performed between March 2013 and June 2014 in 63 women with PC. Patient and sample characteristics are described in Tables 1 and 2, respectively. Prior to study inclusion, all patients had undergone at least two lines of standard intravenous chemotherapy. PIPAC was used as IPC treatment and was performed as described previously [14–17]. At the beginning of each procedure, the Peritoneal Cancer Index (PCI) was determined according to Sugarbaker, based on lesion size and distribution [18]. Prior to the application of chemotherapy (cisplatin at a dose of 7.5 mg/m^2^ body surface in 150 ml 0.9 %-NaCl solution followed by doxorubicin at a dose of 1.5 mg/m^2^ body surface in 50 ml 0.9 %-NaCl solution; see [15]), peritoneal biopsies were taken both for conventional histological analysis and for gene expression testing. The laboratory team was blinded to the clinical outcome. If present, ascites was removed at the same time and ascites volume was documented. PIPAC and PC sampling was repeated every 4 to 6 weeks until progression, death, or unacceptable toxicity. Patients were followed-up until January 2015 or death. Median follow-up was 158 days (range: 9–640 days; interquartile range: 75–310 days).

**Table 1:** Patient characteristics of 63 women with peritoneal cancer undergoing repeated pressurized intraperitoneal aerosol chemotherapy (PIPAC) with cisplatin and doxorubicin

Patient characteristic	Value
Number of patients	63
Age (years; mean ± SD)	62.0 ± 11.3
Previous chemotherapy regimens (median, range)	3 (2–8)
Presence of ascites	35/63 (56.6 %)
Ascites volume (ml; median, range)	150 (10–4500)
PCI (mean ± SD)	17.5 ± 10.0
Serum CA125 (U/ml; mean ± SD)	1590 ± 3753
Primary tumor	
Ovarian cancer	58 (92.1 %)
Endometrial cancer	3 (4.7 %)
Pseudomyxoma peritonei	1 (1.6 %)
Stomach cancer	1 (1.6 %)
Cell type	
Serous papillary adenocarcinoma	37/63 (58.7 %)
Mucinous adenocarcinoma	2/63 (3.2 %)
Other	24/63 (38.1 %)
Number of patients sampled at PIPAC 1 and at least one follow-up PIPAC (2, 3, or 4)	42
Number of patients sampled at PIPAC 1, 2 and 3	28

*SD* standard deviation, *PCI* Peritoneal Cancer Index

**Table 2:** Sample characteristics

Sample characteristic	Value
Total number of analyzed samples	152
Obtained during PIPAC 1 (“untreated”)	53 (34.9 %)
Obtained during PIPAC ≥2 (“treated”)	99 (65.1 %)
Histologically assessed as	
Tumor	136 (89.5 %)
With concurrent ascites	88 (64.7 %)
Without concurrent ascites	48 (35.3 %)
Tumor-free	16 (10.5 %)

### RNA isolation and cDNA synthesis

Total RNA was isolated from snap-frozen tissue samples using the RNeasy Mini RNA isolation kit (Qiagen, Hilden, Germany) following the instructions of the manufacturer. In brief, up to 30 mg of tissue were weighed out in 2-ml-reaction tubes, covered with lysis buffer (RLT, provided with the kit, supplemented with 1 % β-mercaptoethanol), and disrupted/homogenized using a rotor-stator homogenizer (TissueRuptor, Qiagen). The homogenate was applied to the RNeasy spin columns, washed, and finally eluted in water. RNA was quantified using a BioPhotometer (Eppendorf, Hamburg, Germany). cDNA was synthesized using the Maxima First Strand cDNA Synthesis Kit (Life Technologies/Thermo Fisher Scientific) after treatment with RNase-free DNase I (Life Technologies) to eliminate contamination with genomic DNA. Typical yields were (0.56 ± 0.54) μg total RNA per 1 mg tissue (median 0.26, range 0.02–2.41). RNA quality was routinely checked by agarose gel electrophoresis and assessing the integrity of the 28S and 18S rRNA bands.

### Gene panel

In this study, we examined a 22 gene panel (see Additional file 1: Table S1). Specific genes were chosen based on previous literature associating these genes with ovarian cancer carcinogenesis and metastatic promotion [10–13, 19]. A list of corresponding primers, primer sequences, product lengths, and GenBank accession numbers can be found in Additional file 2: Table S2.

### Quantitative real-time PCR

PCR was performed using the Maxima SYBR Green/ROX qPCR Master Mix (Life Technologies) in an ABI 7900HT Fast Real-Time PCR System (Applied Biosystems) in 384-well plates (BIOplastics, Landgraaf, The Netherlands). Reactions (10 μl total volume) consisted of 5 μl 2x Master Mix, 2 μl cDNA, 3 μl primer mix (final primer concentration was 0.3 μM). A two-step cycling protocol was used: 10 min initial denaturation (95 °C) and 40 cycles of 15 s denaturation (95 °C) and 60 s annealing/extension (60 °C). All reactions were carried out in triplicates. Absence of contaminating genomic DNA was confirmed by amplifying cDNA and corresponding amounts of RNA with GAPDH and ACTB primers. Primers were either designed using Primer3 [20] or taken from https://primerdepot.nci.nih.gov. All primers were manually checked against their targets’ GenBank entries and wherever possible, it was made sure that the PCR products spanned exon-exon boundaries. Each product was verified by agarose gel electrophoresis (not shown) and expected melting temperature (OligoCalc) [21]. Specific amplification of products was routinely checked by melting curve analysis after each run. Transcription of 1 μg RNA into cDNA and 100-fold dilution (final) for real-time PCR resulted in cycle threshold (Ct) values of 22.3 ± 3.8 and 23.2 ± 3.7 for the reference genes ACTB and GAPDH, respectively. The difference between the Ct values of the reference genes was 0.9 ± 1.4. Thus, for sample-to-sample comparison of gene expression, the ΔΔCt-method was employed using the mean Ct of both reference genes.

### Immunohistochemistry

Immunohistochemical analysis was performed as described before [22]. The material was routinely fixed in 4 % formaldehyde solution and embedded in paraffin. After slicing into 4-μm-thick sections, the preparations were dewaxed in xylene and then rehydrated. Endogenous peroxidase activity was blocked by 3 % hydrogen peroxide in methanol for 30 min. After a short rinse with phosphate buffered saline (PBS), sections were pre-incubated with avidin-biotin (Vector Laboratories, Peterborough, UK; SP-2001) for 15 min to reduce non-specific background staining. The preparations were covered with normal goat serum for 20 min and then incubated with the primary antibodies (CD44, mouse monoclonal, Diagnostic Biosystems, Pleasanton, CA, dilution 1:2000; CD44v6, abcam, Cambridge, MA, dilution 1:1000; and VEGF, mouse monoclonal, Dako, Denmark, dilution 1:50) for 30 min. Then, the sections were washed with PBS, incubated with biotinylated goat anti-mouse immunoglobulin G (BioGenex, Germany) for 30 min and covered with peroxidase-conjugated streptavidin (Dako). The peroxidase reaction was allowed to proceed for 8 min, with 0.05 % 3,3-diaminobenzidine tetrahydrochloride solution as substrate. Slides were counterstained with hematoxylin. Negative controls were also performed by replacing the primary antibodies with mouse or goat ascites fluid (Sigma-Aldrich, St. Louis, MO).

### Data analysis and statistics

**Fig. 1:**
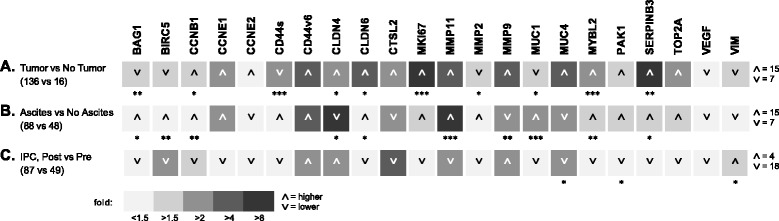
Comparison of mRNA expression levels of a panel of genes between groups of **a** samples histologically assessed as tumor vs. tumor-free; **b** assessed as tumor with concomitant presence of ascites vs. no ascites; and **c** obtained after initial IPC (post, treated) vs. initial sampling before IPC within the first PIPAC procedures (pre, untreated). In each case, the samples were taken before the application of the chemotherapy aerosol. The magnitude of the mean expression level ratios of the groups is indicated by different levels of gray (from <1.5-fold, lightest gray to >8-fold, darkest gray). Arrowheads indicate whether the expression is higher (up) or lower (down) in the first group. The numbers of genes expressed higher/lower are indicated at the far right. Statistical significance of differences between the groups (Mann–Whitney rank sum test) is indicated by asterisks (***, *p* < 0.001; **, *p* < 0.01; *, *p* < 0.05). A color version of the figure is available in Additional file 7

**Fig. 2:**
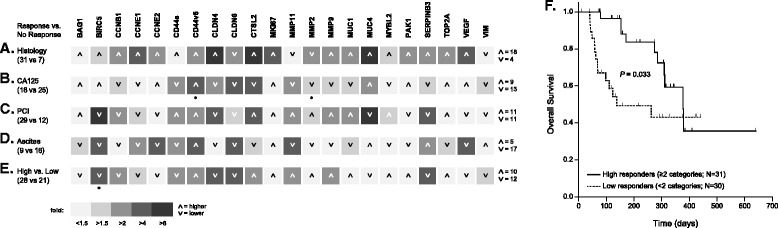
Predictive value of gene expression profiles. Patients were grouped into responders and non-responders judged by histological evaluation of tissue samples (regression; **a**), serum levels of CA125 (decline; **b**), PCI (improved; **c**) or volume of ascites (reduction; **d**). Panel **e** shows patients grouped as high (response in ≥2 categories) and low responders (response in <2 categories). Corresponding samples obtained before the first IPC and histologically confirmed as tumors were analyzed. The magnitude of the mean expression level ratios of the groups is indicated by different levels of gray (from <1.5-fold, lightest gray to >8-fold, darkest gray). Arrowheads indicate whether the expression is higher (up) or lower (down) in the respective response group (or high responders in E, respectively). The numbers of genes expressed higher/lower are indicated at the far right. Statistical significance of differences between the groups (Mann–Whitney rank sum test) is indicated by asterisks (*, *p* < 0.05). Panel **f** shows overall survival of high and low responders (Kaplan-Meier log rank analysis). A color version of the figure is available in Additional file 8

Amplification data were analyzed using the SDS 2.4.1 and RQ Manager 1.2.1 software packages (Applied Biosystems). Clinical data and gene expression data were entered and further processed in Microsoft Excel 2013. Statistical analyses and data visualization was carried out using SigmaPlot 12.5 (Systat Software, San Jose, CA). Gene expression data from our sample collective rarely followed a normal distribution. Thus, non-parametric tests were used. To compare gene expression between groups of samples (such as those in Figs. 1 and 2), the Mann–Whitney U (Wilcoxon) rank sum test was used. For the analysis gene expression changes in specimens drawn from the same individuals over multiple samplings, the following statistical tests were used: Wilcoxon signed rank test, Friedman’s repeated measures analysis of variance (ANOVA) on ranks (Tukey test for pairwise multiple comparisons; in case of normal distribution of the data, one way repeated measures ANOVA was used with the Holm-Sidak method for pairwise multiple comparisons), and two way repeated measures ANOVA (to test for interaction with other variables). The statistical significance of survival curve differences was assessed using the Kaplan-Meier log rank analysis. To assess hazard ratios, the Cox proportional hazards model was employed.

## Results

### Molecular markers differentiate PC tumor tissue from normal peritoneum

Peritoneal biopsies were taken during each PIPAC cycle immediately prior to the application of chemotherapy. In 16 of 152 peritoneal biopsies, no tumor tissue was identified in the histological examination. These tissue samples served as non-tumor controls representing undiseased peritoneum. Tumor and non-tumor samples differed significantly in the expression of 10/22 genes (Fig. 1a): CCNB1, CLDN4, CLDN6, MMP2, MUC1 (all: *p* < 0.05), BAG1, SERPINB3 (all: *p* < 0.01), CD44 (standard variant), MKI67, and MYBL2 (all: *p* < 0.001). As expected, the tumor sample population showed higher expression levels of pro-mitotic genes such as CCNB1 and MYBL2, genes that modulate the host immune response, such as SERPINB3, and the tight-junctions proteins claudin-4 and claudin-6, which are often deregulated in cancers. CD44 isoforms were also found to be deregulated as observed in many types of cancer. Interestingly, the anti-apoptotic gene BAG1 appeared to be slightly diminished (less than 2-fold) in the tumor sample population.

### Ascites-producing tumors have a distinct gene expression profile

We expected that ascites-producing PC differs from non ascites-producing PC on the molecular level and that this difference would potentially be reflected in our gene panel. Indeed, when comparing tumor samples from PC with ascites (88/136; 65 %) and without ascites (48/136; 35.3 %), 10/22 genes showed significantly different expression levels (Fig. 1b). BAG1, CLDN6, SERPINB3 (all: *p* < 0.05), BIRC5, CCNB1, MYBL2 (all: *p* < 0.01), and MMP11 and MUC1 (all: *p* < 0.001) were expressed at higher, and CLDN4 (*p* < 0.05) and MMP9 (*p* < 0.01) were expressed at lower levels in samples from patients with ascites.

### Samples from IPC-treated patients show an overall lower expression of the gene panel

Next, we compared the average gene expression levels in samples from patients who had undergone at least one IPC treatment round (“post”; obtained during second or later PIPAC procedure) to those in samples from IPC-naïve patients (“pre”; obtained during the first PIPAC procedure). We observed an overall lower expression in 18/22 genes (Fig. 1c), although with rare and only weak statistical significance.

### Gene expression profiles prior to IPC and prediction of treatment response

In order to identify predictive molecular markers for treatment response, we compared the gene expression profiles in sample sets obtained during the first PIPAC procedure (i.e. IPC-naïve) from patients classified as responders or non-responders in four response categories. Response to PIPAC treatment was measured by a) histologic regression, b) decline of serum CA125 levels, c) improved PCI, and d) reduction of ascites volume, in each case comparing the status before the initial PIPAC with that at PIPAC 3 (or in few cases, earlier for patients who could not continue treatment cycles due to side effects or death). We found that gene expression patterns before the start of chemotherapy were not predictive of any of these four response categories (Fig. 2a-d). Gene expression patterns were comparable in pre-IPC tumor samples with or without subsequent clinical response. The same was true when the samples obtained prior to the first PIPAC treatment were stratified based on a composite of response criteria (low responders = response in <2 of the four categories described above versus high responders = response in ≥2 categories) (Fig. 2e). That the response criteria are clinically valid and correspond to the oncological outcome was demonstrated by a significantly diminished overall survival of the low responders (*p* = 0.033) with a hazard ratio (HR) of 2.37 (95 % confidence interval [CI] 1.05–5.38; *p* = 0.037).

### Differential gene expression during IPCs predicts treatment response and survival

To assess the effect of PIPAC treatment on individual patients, we looked at gene expression changes over the course of up to six PIPAC treatments. Additional file 3: Figure S1 shows “spaghetti plots” for all 22 genes in our gene panel. Expression patterns of individual genes in our patient sample are quite heterogeneous. Some vary only moderately (at most approx. 4-fold) across patients and time points (e.g. BAG1, MMP2, VIM), others are very heterogeneously expressed, both in magnitude and over time (e.g. CLDN4 and 6, MUC1 and 4, SERPINB3).

**Fig. 3:**
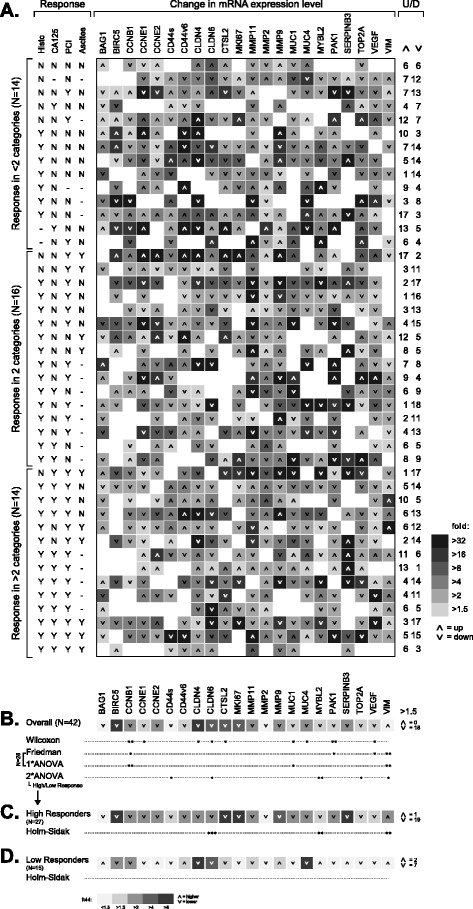
Heat map of gene expression changes in patient samples throughout IPC cycles **a** and of IPC-induced overall gene expression change **b** and in subset of high **c** and low response patients **d** judged by clinical response categories. **a** Each row represents a patient for whom an IPC-naïve tumor sample and at least one sample from PIPAC cycles 2 or 3 was available (*n* = 44), and each column corresponds to a specific gene. Patients (rows) were grouped according to treatment response as judged by histologic regression, decline of serum CA125 levels, improved PCI, and reduction of ascites volume as indicated in the columns to the left of the heat map (Y = response, *N* = no response, − = not applicable/data missing). **b** Statistical significance of expression change for individual genes was assessed by the Wilcoxon signed rank test, Friedman ANOVA on ranks (or one way [1*] ANOVA in case of normally distributed data; note that only data from a subset of 28 patients was considered; see text), and two way (2*) ANOVA using high/low clinical responders as additional factor. **c**, **d** Expression changes in samples of high and low responders and statistical significance assessed by the Holm-Sidak method of multiple pairwise comparison within the two way ANOVA described in B. **a**-**d** The magnitude of expression change is reflected by different levels of gray intensity (see legends); arrowheads indicate whether gene expression goes up or down in the course of IPC. The numbers of up-/downregulated genes are indicated at the far right. Statistical significance is indicated by asterisks (***, *p* < 0.001; **, *p* < 0.01; *, *p* < 0.05). A color version of the figure is available in Additional file 9

To further analyze the expression data longitudinally, we encoded the expression change of each gene for all patients where samples at different time points were available (*N* = 44; in all cases the tumor of origin was ovarian cancer) as a heat map (Fig. 3a). Expression change was calculated as the fold-change from before the initial treatment (*N* = 42), to the expression levels in the sample obtained during the third PIPAC procedure (*N* = 31), or when this sample was not available from those during the fourth (*N* = 1) or second (*N* = 10) PIPAC procedure; additionally, two cases were included where only samples from the second and third PIPAC procedure were available, but not from the initial procedure (*N* = 2). In Fig. 3a, patients (each row corresponds to an individual patient) are grouped by clinical response (as detailed in the columns to the left of the heat map). Of note, when looking at (the color version of) Fig. 3a, treatment response appears to be associated with general downregulation of the whole gene panel induced by the treatment. In the low response group (response in <2 categories), ≥1.5-fold upregulation or downregulation occurred in 107 and 114 analyses, respectively. In contrast, ≥1.5-fold upregulation or downregulation in the high response groups (response in ≥2 categories) occurred in 175 and 308 analyses, respectively (*p* = 0.003; two-tailed, Fisher’s exact test).

A more sophisticated statistical analysis of these data is shown in Fig. 3b–d. Figure 3b shows the average expression level changes induced by IPC and demonstrates that treatment leads to an overall downregulation of the whole gene panel. Using a cutoff of at least 1.5-fold change in expression level, 18/22 genes were found downregulated and none upregulated. The differences in expression before and after treatment were statistically significant (Wilcoxon signed rank test) in case of CCNB1, PAK1 (both: *p* < 0.01), CCNE1, CLDN4, CLDN6, CTSL2, MUC1, MUC4, and VEGF (all: *p* < 0.05). Repeating this analysis on a subset of cases (*N* = 28) where three consecutive samples (PIPAC 1, 2, and 3) were available, Friedman repeated measures ANOVA on ranks (one way repeated measures ANOVA in select cases where data followed a normal distribution), CCNB1, MUC1, PAK1, and VEGF downregulation was confirmed as statistically significant, and additionally revealing an upregulation of VIM (*p* < 0.01) in this subset of patients. In all cases, a statistically significant difference was present between the initial sample (“untreated”) and that obtained during the third PIPAC (i.e. having undergone two rounds of IPC).

In addition, a statistically significant interaction between treatment induced change of gene expression and response judged by clinical response categories was found for MYBL2 (*p* < 0.01), CD44s, CLDN6, TOP2A, and VIM (all: *p* < 0.05). In the subgroup of high responders (Fig. 3c), statistically significant downregulation of CLDN6 (*p* < 0.001) and MYBL2 (*p* < 0.01) was found, while VIM was upregulated (*p* < 0.01). In the low response group (Fig. 3d), no significant differences were present and the effect of overall IPC-induced downregulation of the gene panel was lost.

**Fig. 4:**
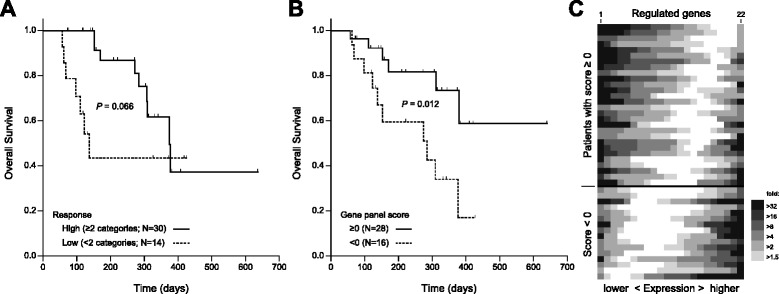
Prognostic value of gene panel expression changes induced by IPC. Overall survival of patients stratified as high and low responders **a** is juxtaposed to that of patients with a gene panel score ≥ 0 or < 0 **b**. The heat map shown in Fig. 3a has been reformatted **c**. Each row represents a patient (*n* = 44) with individual genes sorted from greatest downregulation (*left*) to greatest upregulation (*right*); the magnitude of gene expression change is indicated (from <1.5-fold, white, to >32-fold, darkest). Rows are sorted by gene panel score, highest (top) to lowest (bottom). A color version of the figure is available in Additional file 10

We hypothesized that the degree of IPC-induced up- and downregulation of the whole 22-gene panel can serve as a predictor of patient outcome on par or better than the clinical response categories histology, CA125, PCI and/or ascites. To test this hypothesis, we calculated a “gene panel score” for each patient (*N* = 44) from the data shown in Fig. 3a. The score was calculated as a weighted sum across the genes, downregulation counting as positive, upregulation as negative. The weighting factors for changes <1.5-fold and changes ≥1.5, 2, 4, 8, 16, and 32-fold in gene expression were 0, 0.5, 1, 2, 3, 4, and 5, respectively. The overall survival of the 44 patients included in this analysis, grouped as low and high responders, is shown in Fig. 4a. Note that the survival curves for both groups are similar to those for the superset of 61 patients shown in Fig. 2f, but their difference is not quite statistically significant (*p* = 0.066; Cox regression: HR = 2.5, 95 %CI 0.9–6.8, *p* = 0.075). When defining a cutoff of zero for the score, and thus comparing overall survival between patients with a score ≥0 to those with a score <0, survival of the latter group is significantly diminished (*p* = 0.012; Cox regression: HR = 3.4, 95 %CI 1.2–9.5, *p* = 0.018) (Fig. 4b). Thus, the gene panel score appears superior to our combination of clinical response categories. A graphical representation of the differential gene expression data in the patients according to gene panel score is shown in Fig. 4c. To arrive at this figure, the heat map shown in Fig. 3a has been transformed, first by sorting rows (patients) top to bottom with decreasing gene panel score, and within rows by sorting by magnitude of gene expression change, greatest downregulation (left) to greatest upregulation (right).

Additional file 4: Figure S2 shows Kaplan-Meier log rank analysis of patient overall survival (*N* = 42) grouped by ≥1.5-fold upregulation, ≥1.5-fold downregulation, or <1.5-fold change for each of the 22 genes in the gene panel. Statistically significant differences in survival were identified for BAG1 (no change vs. downregulation) and CLDN6 (up- vs. downregulation), both at *p* < 0.05.

### Immunohistochemistry validated results obtained on the mRNA level

In a subset of PC tumor samples selected based on large difference in expression level on the mRNA level, we analyzed the protein expression of CD44, CD44v6, and VEGF by immunohistochemistry (based on established in-house methods and availability of antibodies). Semiquantitative analysis of CD44, CD44v6, and VEGF expression confirmed expression changes during intraperitoneal chemotherapy, i.e. gain or loss of protein expression. Additional file 5: Figure S3 demonstrates loss/gain of CD44, CD44v6, and VEGF expression when comparing samples taken before the first IPC and samples taken during subsequent PIPAC procedures.

## Discussion

In the present study we found that molecular markers differentiate between PC tumor tissue and tumor-free peritoneum as well as between ascites-producing and non ascites-producing PC. PC samples taken prior to PIPAC had no predictive value, but gene expression changes during repeated IPC were associated with clinical treatment response. A molecular subtype characterized by overall downregulation of the whole gene panel predicted overall patient survival. Thus, the analysis of consecutive PC tumor samples obtained during repeated cycles of PIPAC allowed us to document response of PC to IPC on the molecular level and to correlate these findings with response parameters and clinical outcome.

One of the main finding of this study is that the expression changes of the 22 gene panel are associated with disease outcome. Specifically, when we looked at the interaction between clinical responses (such as histologic regression, improved PCI, declining CA125 serum levels, and reduced ascites production) and molecular response, we observed an even more pronounced overall downregulation of the gene panel (with the exception of VIM) in patients showing clinical improvement versus those who did not. In the first group, three genes showed statistically significant changes: CLDN6 and MYBL2 (both downregulated) and VIM (upregulated), whereas CLDN6 and VIM were downregulated and upregulated, respectively, in the latter group.

MYBL2 was specifically downregulated in the high response group. Ren et al. reported that, in colorectal cancer, MYBL2 was significantly overexpressed and inversely correlated with disease-free survival [23]. It has further been described as a putative biomarker found upregulated in cervical cancer [24]. While the role of MYBL2 in ovarian cancer and derived entities is not well established, our data would indicate that it acts similarly in PC of ovarian cancer origin. In line with this is that MYBL2 is located on a chromosomal area (20q) recently identified by in silico chromosomal clustering of genes displaying altered expression patterns in ovarian cancer [25].

New prognostic markers for patients with PC are needed to refine therapy and prognosis estimation in this precarious patient group. Consistent with previous studies, clinical and histopathological features such as histologic regression, decline of serum CA125 levels, improved PCI, and reduction of ascites volume during the course of IPC were associated with prognosis [15, 26]. Here, we demonstrate that our gene panel and the score derived from its expression level changes throughout repeated IPC treatments represents a prognostic marker that is superior to clinical and histopathological features described above, individually or in combination. It is, however, a limitation of our study that we used a pre-selected gene panel instead of a whole genome expression analysis. Thus, we cannot rule out that the positive findings in this study only represent a subset of relevant genes, which are differentially regulated by IPC (and potentially specifically by PIPAC). Further studies using whole genome analyses and larger sample sets may expand our preliminary findings.

As expected, we found that gene expression patterns are different in PC tumor samples compared to normal, undiseased peritoneum with a number of genes differentially regulated: BAG1, CCNB1, CD44, CLDN4 and 6, MMP2, MKI67, MUC1, MYBL2, and SERPINB3. Given the properties of their protein products, this finding is consistent with the hypothesis that malignant transformation of peritoneum into PC is associated with deregulation of three mechanisms [27]: (a) growth and proliferation via BAG1, CCNB1, MKI67, and MYBL2, (b) cell adhesion and forced migration via CD44 standard, MUC1, CLDN4, and CLDN6, followed by increased local invasiveness through upregulation of MMP2, and (c) VEGF-mediated angiogenesis. Deregulation of the expression of BAG1, CCNB1, MKI67, and MYBL2 was documented in PC biopsies, suggesting that growth and cell proliferation pathways are disturbed, a feature of malignant transformation in ovarian and peritoneal cancer that has been widely documented in the literature [10–13, 19, 27]. Loss of cell adhesion via deregulation of CD44, CLDN4, and CLDN6, followed by increased migration and invasive capabilities through upregulation of MMP2 describes a pattern consistent with localized ovarian cancer cells transforming into metastasizing PC cells, and expands previous data showing that deregulation of proliferation and cell adhesion is a feature of malignant transformation in ovarian and peritoneal cancer [10–13, 19, 28]. Tjhay et al., for instance, have shown that disseminated ovarian tumors in the peritoneum contain highly enriched CD44v6-positive cancer cells and an increased number of CD44v6-positive cancer cells in primary tumors was associated with a shortened overall survival in stage III-IV ovarian cancer [29]. In a xenograft model, Haria et al. found that DLX4, a transcription factor encoded by a homeobox gene, induced expression of CD44 in ovarian tumor cells, and inhibition of CD44 abrogated the ability of DLX4 to stimulate tumor-mesothelial cell interactions [30]. The fact that CD44 drives inflammatory signaling in PC is also consistent with our finding of differential regulation of SERPINB3, associated with host-immune-response [31]. Furthermore, strong expression of SERPINB3 protein was characterized as a prognostic factor for platinum resistance and for poor progression-free survival [32], providing an alternative or additional explanation for the increased expression of SERPINB3 in our patients who all had undergone several systemic chemotherapies before recruitment for PIPAC.

Besides gene expression differences between PC samples and tumor-free peritoneum, we also found that ascites-producing PC has a distinct gene expression profile compared to non-ascites producing PC. Specifically, 10/22 genes were differently expressed, namely BAG1, BIRC5, CCNB1, CLDN4 and 6, MMP9 and 11, MUC1, MYBL2, and SERPINB3. This finding of a gene signature for ascites-producing PC is consistent with previous data analyzing differences in gene expression between PC with miliary and non-miliary peritoneal spread [33]. In this study, Auer et al. analyzed tumor tissues and ascites from 23 chemotherapy-naive patients by RNA-sequencing and flow cytometry. Pathway analysis of the differentially expressed genes in ascites yielded 11 deregulated pathways including MMP9 as deregulated key player. This is consistent with our finding of MMP9 being deregulated in ascites-producing PC.

As for inclusion criteria, only patients who had undergone previous platinum-containing chemotherapy were treated with PIPAC. Thus, all patients can be considered platinum-resistant. The reason for using cisplatin as one of the intraabdominal drugs is that the chemotherapy concentration is significantly higher after local application than after systemic application and may thus overcome platinum resistance by local drug escalation.

## Conclusion

In summary, we documented a molecular response to IPC in PC tumor samples obtained during repeated consecutive cycles of PIPAC with cisplatin and doxorubicin. These changes correlated with clinical outcome and could be used to predict overall survival. Thus, measuring gene expression changes during the time window of treatment may be useful for refining individual treatment and prognosis estimations.

## Additional files

Additional file 1: Table S1. Gene panel. (DOC 31 kb) Additional file 2: Table S2. Genes, primer sequences, and amplicon data. (DOC 58 kb) Additional file 3: Figure S1. Gene expression shown as “spaghetti plots” for each gene in the gene panel in peritoneal samples obtained prior to the administration of the chemotherapeutics during up to six IPC treatment cycles at approximately 6-weeks intervals (1, IPC-naïve, *n* = 53; 2, *n* = 49; 3, *n* = 38; 4, *n* = 7; 5, *n* = 3, 6; *n* = 2). Dots represent individual samples; lines connect samples from individual patients, taken at consecutive IPCs. Expression is shown as the difference in the cycle threshold (Ct) value between the respective gene and the mean Ct of the reference genes (ACTB, GAPDH); higher values correspond to stronger expression. Gene symbols are indicated in the panels. (PDF 646 kb) Additional file 4: Figure S2. Overall survival vs. level of gene expression change in peritoneal samples of patients treated with PIPAC. Inside panels: Gene symbol, lower left corner; numbers of patients with upregulated/unchanged/downregulated gene expression (top to bottom), upper right corner; Kaplan-Meier log rank p-value, lower right corner. Statistical significance is indicated by brackets (*, *p* < 0.05). Cox proportional hazard regression resulted in HR = 5.1 (95 %CI 1.3–19.0, *p* = 0.017) for BAG1 and HR = 4.1 (95 %CI 1.2–13.6, *p* = 0.02) for CLDN6. (PDF 384 kb) Additional file 5: Figure S3. Exemplary images of haemotoxylin/eosin (H&E, left columns) and immunohistochemical (right columns) stainings of tissue sections from patients obtained during initial and subsequent PIPAC treatments (before = IPC-naïve, after = PIPAC 2 or 3) using antibodies to CD44 (all isoforms), CD44 variant 6 (CD44v6), and VEGF. Each row represents samples from an individual patient (note, rows 1 and 4, and rows 3 and 6 are from the same patient, respectively, but not necessarily from the same after-PIPAC time point). Scale bars, 100 μm. (PDF 1917 kb) Additional file 6: Raw data (clinical and qPCR data). (XLSX 90 kb) Additional file 7: Color version of Figure 1. (EPS 860 KB) Additional file 8: Color version of Figure 2. (EPS 944 KB) Additional file 9: Color version of Figure 3. (EPS 1.90 MB) Additional file 10: Color version of Figure 4. (EPS 864 KB)

## Abbreviations

HIPEC: Hyperthermic intraperitoneal chemoperfusion
HPMCs: Human peritoneal mesothelial cells
IHC: Immunohistochemistry
IPC: Intraperitoneal chemotherapy
PC: Peritoneal carcinomatosis
PCI: Peritoneal carcinomatosis index
PIPAC: Pressurized intraperitoneal aerosol chemotherapy.
