# Supplementary material for: Dynamic changes of tumor gene expression during repeated pressurized intraperitoneal aerosol chemotherapy (PIPAC) in women with peritoneal cancer
Source: BMC Cancer. 2016 Aug 19;16:654. doi: 10.1186/s12885-016-2668-4 (PMC4992274; doi:10.1186/s12885-016-2668-4)
Supplement: Additional file 1: Table S1. — Gene panel. (DOC 31 kb) [file 12885_2016_2668_MOESM1_ESM.doc]

**Supplementary Table S1.** Gene panel.

| **Growth/Proliferation** |
| --- |
| BAG1, BIRC5, CCNB1, CCNE1, CCNE2, MKI67, MYBL2, TOP2A |
| **Apoptosis, Anti-Apoptosis** |
| BAG1, BIRC5, MMP2, MMP9, PAK1, VEGF |
| **Migration/Invasion** |
| CD44, CTSL2, MMP2, MMP9, MMP11, PAK1, VIM |
| **Cell Adhesion / Cell Junction Organization** |
| CD44 (s, v6), MUC1, MUC4, CLDN4, CLDN6 |
| **Host Immune Response** |
| SERPINB3 |
| **Angiogenesis** |
| VEGFA |
| **Reference** |
| ACTB, GAPDH |

Gene symbols: **ACTB** (β-actin), **BAG1** (BCL2-associated athanogene), **BIRC5** (baculoviral IAP repeat containing 5; apoptosis inhibitor 4), **CCNB1** (cyclin B1), **CCNE1/2** (cyclin E1/2), **CD44** (CD44 molecule, standard isoform and variant 6), **CLDN4/6** (claudin-4/-6), **CTSL2** (cathepsin V, cathepsin L2), **GAPDH** (glyceraldehyde 3-phosphate dehydrogenase), **MKI67** (marker of proliferation Ki-67), **MMP2** (matrix metallopeptidase 2, Gelatinase A), **MMP9** (matrix metallopeptidase 9, Gelatinase B), **MMP11** (matrix metallopeptidase 11, stromelysin 3), **MUC1/4** (mucin 1/4, cell surface associated), **MYBL2** (v-myb avian myeloblastosis viral oncogene homolog-like 2), **PAK1** (p21 protein [Cdc42/Rac]-activated kinase 1), **SERPINB3** (serpin peptidase inhibitor, clade B [ovalbumin], member 3), **TOP2A** (topoisomerase [DNA] II alpha 170kDa), **VEGF** (vascular endothelial growth factor A), **VIM** (vimentin).
