# Supplementary material for: Dynamic changes of tumor gene expression during repeated pressurized intraperitoneal aerosol chemotherapy (PIPAC) in women with peritoneal cancer
Source: BMC Cancer. 2016 Aug 19;16:654. doi: 10.1186/s12885-016-2668-4 (PMC4992274; doi:10.1186/s12885-016-2668-4)
Supplement: Additional file 2: Table S2. — Genes, primer sequences, and amplicon data. (DOC 58 kb) [file 12885_2016_2668_MOESM2_ESM.doc]

**Supplementary Table S2.** Genes, primer sequences, and amplicon data.

| **Symbol^a^** | **Name** | **Forward (5’→3’)** | **Reverse (5’→3’)** | **Accession No.** | **Length (bp)** | **T_m_  (°C)** |
| --- | --- | --- | --- | --- | --- | --- |
| ACTB | beta-actin | AGCAAGCAGGAGTATGACG | GAAAGGGTGTAACGCAACT | NM_001101 | 90 | 80.2 |
| GAPDH | glyceraldehyde-3-phosphate dehydrogenase | ACAGTCAGCCGCATCTTCTT | ACGACCAAATCCGTTGACTC | NM_002046 | 94 | 82.5 |
| BAG1 | BCL2-associated athanogene | GAAATGGAAACACCGTTGTC | AACCTCTTCCTGTGGACTGT | NM_004323 | 90 | 76.4 |
| BIRC5 | survivin | TGCAACCGCCTAGACTTTCT | TCACAACCCTTCCCAGACT | NM_001168 | 90 | 76.2 |
| CCNB1 | cyclin B1 | ATGGTCTCCTGCAACAACCT | CGGGAAGTCACTGGAAACAT | NM_031966 | 86 | 76.0 |
| CCNE1 | cyclin E1 | GAAATGGCCAAAATCGACAG | TCTTTGTCAGGTGTGGGGA | NM_001238 | 110 | 82.3 |
| CCNE2 | cyclin E2 | ACCTCATTATTCATTGCTTCCAA | TCTTCACTGCAAGCACCATC | NM_057749 | 92 | 74.5 |
| CD44 (s) | CD44 molecule (Indian blood group) | GGTTACATCTTTTACACCTTTTCTAC | GAATGTGTCTTGGTCTCTGGTAG | NM_000610 | 111 | 79.6 |
| CD44 (v6) | CD44 molecule (Indian blood group) | GGTTACATCTTTTACACCTTTTCTAC | TAGGAGTTGCCTGGATGGTAG | NM_000610 | 109 | 80.2 |
| CLDN4 | claudin-4 | TAACTGCTCAACCTGTCCCC | ATAAAGCCAGTCCTGATGCG | NM_001305 | 103 | 81.9 |
| CLDN6 | claudin-6 | CCCTTATCTCCTTCGCAGTG | ATGCTGTTGCCGATGAAAG | NM_021195 | 158 | 85.3 |
| CTSL2 | cathepsin L2 | CTACGTGACGCCAGTGAAGA | TTCCGGAACATCTGTCCTTC | NM_001333 | 90 | 78.4 |
| MKI67 | antigen identified by monoclonal antibody Ki-67 | CTTTGGGTGCGACTTGACGA | GGCCAGAAGCAAATTTACAAC | NM_001145966 | 90 | 82.3 |
| MMP2 | matrix metallopeptidase 2 | ATGCCGCCTTTAACTGGAG | GGGAAGCCAGGATCCATTTT | NM_004530 | 103 | 75.9 |
| MMP9 | matrix metallopeptidase 9 | ACGACGTCTTCCAGTACCGA | TTGGTCCACCTGGTTCAACT | NM_004994 | 95 | 80.7 |
| MMP11 | matrix metallopeptidase 11 (stromelysin 3) | CCTGGAGGCTGCAACATACC | TACAATGGCTTTGGAGGATAGCA | NM_005940 | 90 | 80.5 |
| MUC1 | mucin 1 | AGACGTCAGCGTGAGTGATG | GACAGCCAAGGCAATGAGATAG | NM_002456 | 139 | 85.1 |
| MUC4 | mucin 4 | TCACTCTGGAGATTCTAGCAAGAAGT | ATTGCAATGGCAGACCACAGT | NM_018406 | 83 | 78.0 |
| MYBL2 | v-myb myeloblastosis viral oncogene homolog (avian)-like 2 | TGGATGAGGATGTGAAGCTG | GGTGAGGCTGGAAGAGTTTG | NM_002466 | 89 | 79.1 |
| PAK1 | P21 protein (Cdc42/Rac)-activated kinase 1 | AGGAGGAGGAGCCGAGAG | TGGTATTTCTCATCGGAGGG | NM_002576 | 120 | 82.2 |
| SERPINB3 | serpin peptidase inhibitor, clade B (ovalbumin), member 3 | AACACCACAGGAAAAGCTGC | AGAGCTTGTTGGCGATCTTC | NM_006919 | 130 | 76.4 |
| TOP2A | topoisomerase (DNA) II alpha | GCCCTCAAGAAGATGGTGTG | TGCCAATGTAGTTTGTTTCTTTG | NM_001067 | 104 | 74.2 |
| VEGFA | vascular endothelial growth factor A | CCTTGCTGCTCTACCTCCAC | GCAGTAGCTGCGCTGATAGA | NM_003376 | 119 | 82.3 |
| VIM | vimentin | GAAATTGCAGGAGGAGATGC | GCAAAGATTCCACTTTGCGT | NM_003380 | 122 | 79.2 |

^a^ Specific variants detected by the primer pairs are indicated in parenthesis.
