# Supplementary material for: Dynamic changes of tumor gene expression during repeated pressurized intraperitoneal aerosol chemotherapy (PIPAC) in women with peritoneal cancer
Source: BMC Cancer. 2016 Aug 19;16:654. doi: 10.1186/s12885-016-2668-4 (PMC4992274; doi:10.1186/s12885-016-2668-4)
Supplement: Additional file 3: Figure S1. — Gene expression shown as “spaghetti plots” for each gene in the gene panel in peritoneal samples obtained prior to the administration of the chemotherapeutics during up to six IPC treatment cycles at approximately 6-weeks intervals (1, IPC-naïve, n = 53; 2, n = 49; 3, n = 38; 4, n = 7; 5, n = 3, 6; n = 2). Dots represent individual samples; lines connect samples from individual patients, taken at consecutive IPCs. Expression is shown as the difference in the cycle threshold (Ct) value between the respective gene and the mean Ct of the reference genes (ACTB, GAPDH); higher values correspond to stronger expression. Gene symbols are indicated in the panels. (PDF 646 kb) [file 12885_2016_2668_MOESM3_ESM.pdf]

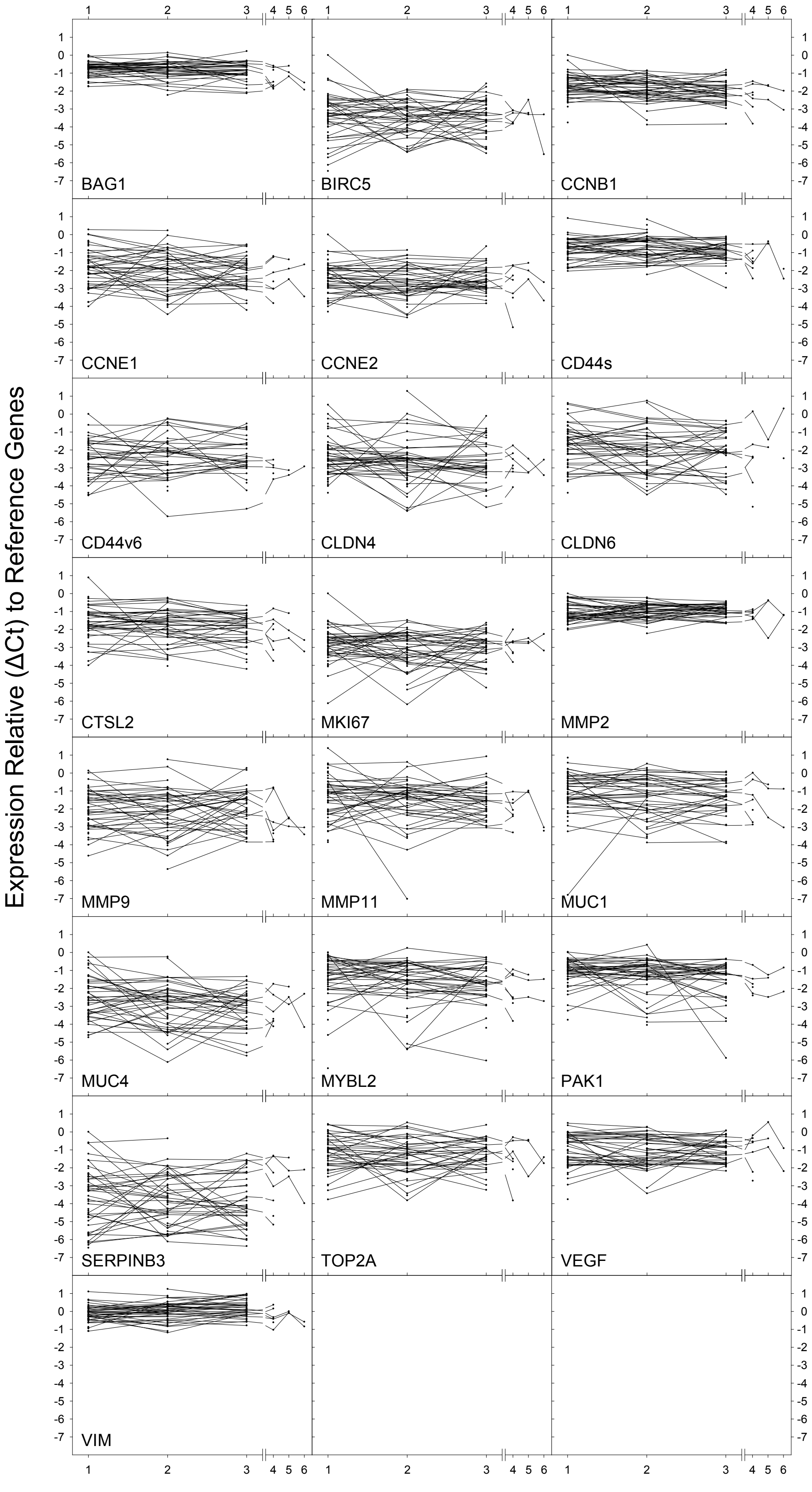

Supplementary Figure S1. Gene expression shown as "spaghetti plots" for each gene in the gene panel in peritoneal samples obtained prior to the administration of the chemotherapeutics during up to six IPC treatment cycles at approximately 6-weeks intervals (1, IPC-naïve, n=53; 2, n=49; 3, n=38; 4, n=7; 5, n=3, 6; n=2). Dots represent individual samples; lines connect samples from individual patients, taken at consecutive IPCs. Expression is shown as the difference in the cycle threshold (Ct) value between the respective gene and the mean Ct of the reference genes (ACTB, GAPDH); higher values correspond to stronger expression. Gene symbols are indicated in the panels.
