# Supplementary material for: Dynamic changes of tumor gene expression during repeated pressurized intraperitoneal aerosol chemotherapy (PIPAC) in women with peritoneal cancer
Source: BMC Cancer. 2016 Aug 19;16:654. doi: 10.1186/s12885-016-2668-4 (PMC4992274; doi:10.1186/s12885-016-2668-4)
Supplement: Additional file 4: Figure S2. — Overall survival vs. level of gene expression change in peritoneal samples of patients treated with PIPAC. Inside panels: Gene symbol, lower left corner; numbers of patients with upregulated/unchanged/downregulated gene expression (top to bottom), upper right corner; Kaplan-Meier log rank p-value, lower right corner. Statistical significance is indicated by brackets (*, p < 0.05). Cox proportional hazard regression resulted in HR = 5.1 (95 %CI 1.3–19.0, p = 0.017) for BAG1 and HR = 4.1 (95 %CI 1.2–13.6, p = 0.02) for CLDN6. (PDF 384 kb) [file 12885_2016_2668_MOESM4_ESM.pdf]

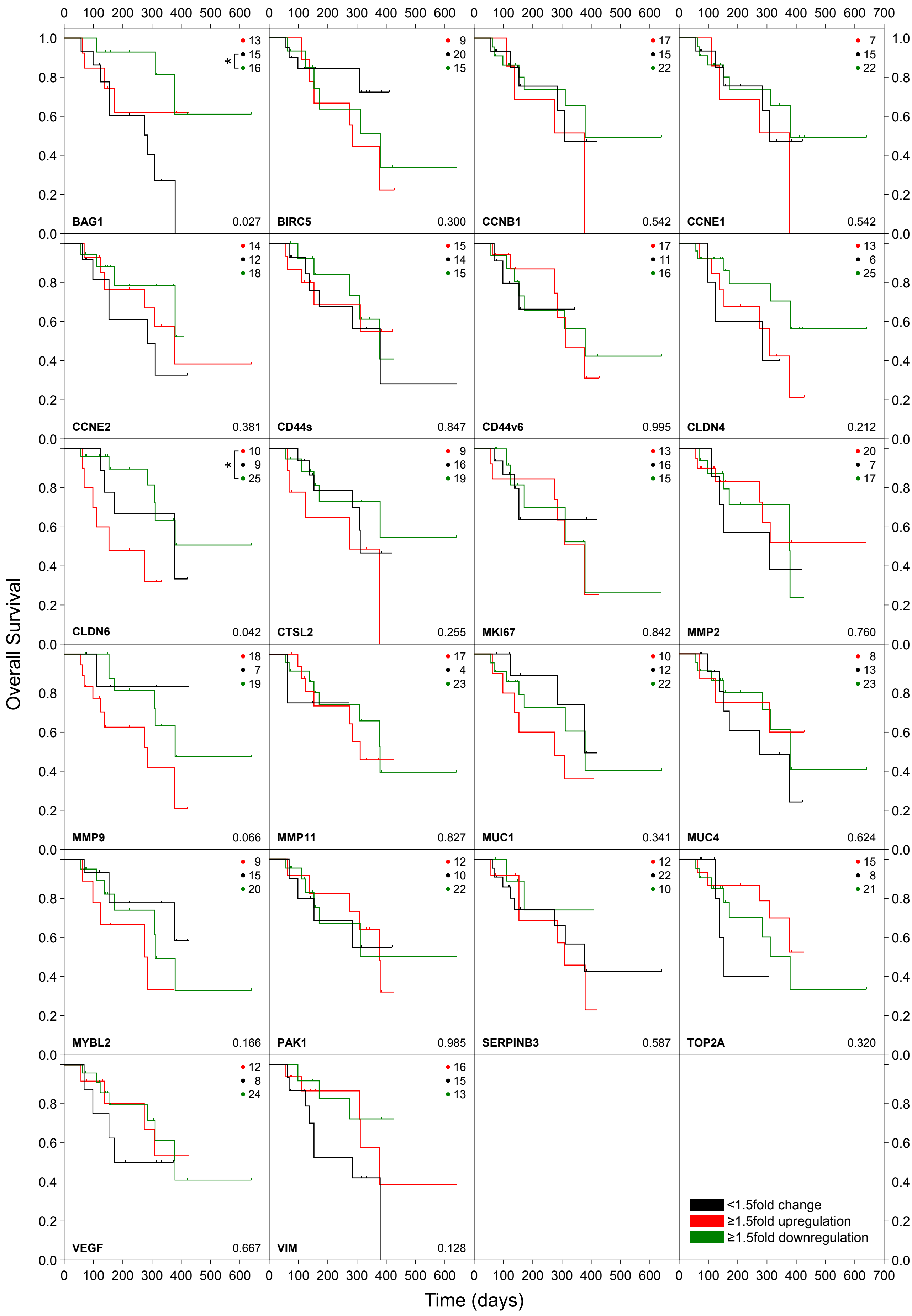

Supplementary Figure S2. Overall survival vs. level of gene expression change in peritoneal samples of patients treated with PIPAC. Inside panels: Gene symbol, lower left corner; numbers of patients with upregulated/unchanged/downregulated gene expression (top to bottom), upper right corner; Kaplan-Meier log rank p-value, lower right corner. Statistical significance is indicated by brackets (*, $p<0.05$). Cox proportional hazard regression resulted in HR=5.1 (95%CI 1.3-19.0, p=0.017) for BAG1 and HR=4.1 (95%CI 1.2-13.6, p=0.02) for CLDN6.
