# Supplementary material for: Dynamic changes of tumor gene expression during repeated pressurized intraperitoneal aerosol chemotherapy (PIPAC) in women with peritoneal cancer
Source: BMC Cancer. 2016 Aug 19;16:654. doi: 10.1186/s12885-016-2668-4 (PMC4992274; doi:10.1186/s12885-016-2668-4)
Supplement: Additional file 5: Figure S3. — Exemplary images of haemotoxylin/eosin (H&E, left columns) and immunohistochemical (right columns) stainings of tissue sections from patients obtained during initial and subsequent PIPAC treatments (before = IPC-naïve, after = PIPAC 2 or 3) using antibodies to CD44 (all isoforms), CD44 variant 6 (CD44v6), and VEGF. Each row represents samples from an individual patient (note, rows 1 and 4, and rows 3 and 6 are from the same patient, respectively, but not necessarily from the same after-PIPAC time point). Scale bars, 100 μm. (PDF 1917 kb) [file 12885_2016_2668_MOESM5_ESM.pdf]

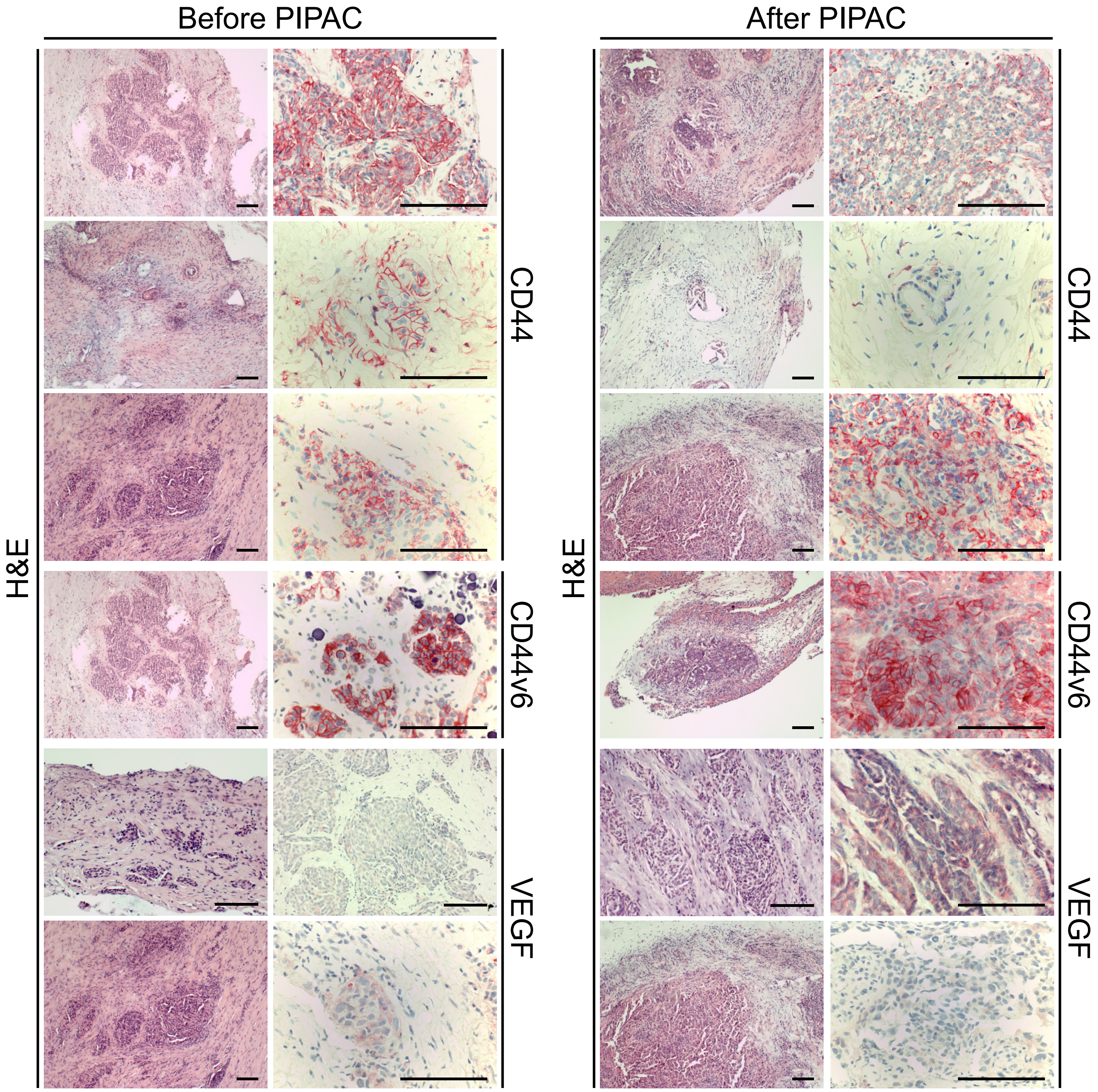

Supplementary Figure S3. Exemplary images of haemotoxylin/eosin (H&E, left columns) and immunohistochemical (right columns) stainings of tissue sections from patients obtained during initial and subsequent PIPAC treatments (before = IPC-naïve, after = PIPAC 2 or 3) using antibodies to CD44 (all isoforms), CD44 variant 6 (CD44v6), and VEGF. Each row represents samples from an individual patient (note, rows 1 and 4, and rows 3 and 6 are from the same patient, respectively, but not necessarily from the same after-PIPAC timepoint). Scale bars, 100 µm.
